# 2,5-Dichloro-*N*-(4-meth­oxy­phen­yl)benzensulfonamide

**DOI:** 10.1107/S1600536811000936

**Published:** 2011-01-12

**Authors:** Islam Ullah Khan, Sajida Bibi, Irfana Mariam, Shahzad Sharif, Sung Kwon Kang

**Affiliations:** a Materials Chemistry Laboratory, Department of Chemistry, Government College University, Lahore 54000, Pakistan; b Department of Chemistry, Chungnam National University, Daejeon 305-764, Republic of Korea

## Abstract

In the title compound, C_13_H_11_Cl_2_NO_3_S, the dihedral angle between the benzene rings is 74.37 (3)°. In the crystal, inter­molecular N—H⋯O hydrogen bonds link the mol­ecules into chains along the *b* axis.

## Related literature

For our previous studies on sulfonamide derivatives, see: Khan *et al.* (2010 ▶); Sharif *et al.* (2010 ▶). For background to the pharmacological uses of sulfonamides, see: Korolkovas (1988 ▶); Mandell & Sande (1992 ▶).
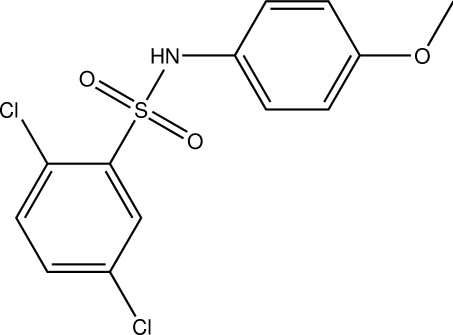


## Experimental

### 

#### Crystal data

C_13_H_11_Cl_2_NO_3_S
*M* *_r_* = 332.19
Monoclinic, 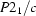

*a* = 13.1599 (4) Å
*b* = 7.8179 (2) Å
*c* = 14.4830 (5) Å
β = 110.566 (1)°
*V* = 1395.09 (7) Å^3^
*Z* = 4
Mo *K*α radiation
μ = 0.62 mm^−1^
*T* = 296 K
0.25 × 0.17 × 0.12 mm

#### Data collection

Bruker APEXII CCD diffractometer
13132 measured reflections
3456 independent reflections
2690 reflections with *I* > 2σ(*I*)
*R*_int_ = 0.030

#### Refinement

*R*[*F* ^2^ > 2σ(*F* ^2^)] = 0.039
*wR*(*F* ^2^) = 0.108
*S* = 1.06
3456 reflections
186 parameters
H atoms treated by a mixture of independent and constrained refinement
Δρ_max_ = 0.46 e Å^−3^
Δρ_min_ = −0.31 e Å^−3^

### 

Data collection: *APEX2* (Bruker, 2007 ▶); cell refinement: *SAINT* (Bruker, 2007 ▶); data reduction: *SAINT*; program(s) used to solve structure: *SHELXS97* (Sheldrick, 2008 ▶); program(s) used to refine structure: *SHELXL97* (Sheldrick, 2008 ▶); molecular graphics: *ORTEP-3 for Windows* (Farrugia, 1997 ▶); software used to prepare material for publication: *WinGX* (Farrugia, 1999 ▶).

## Supplementary Material

Crystal structure: contains datablocks global, I. DOI: 10.1107/S1600536811000936/jh2254sup1.cif

Structure factors: contains datablocks I. DOI: 10.1107/S1600536811000936/jh2254Isup2.hkl

Additional supplementary materials:  crystallographic information; 3D view; checkCIF report

## Figures and Tables

**Table 1:** Hydrogen-bond geometry (Å, °)

*D*—H⋯*A*	*D*—H	H⋯*A*	*D*⋯*A*	*D*—H⋯*A*
N12—H12⋯O19^i^	0.78 (2)	2.50 (3)	3.267 (2)	168 (2)

Symmetry code: (i) 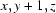.

**Fig. 1.**
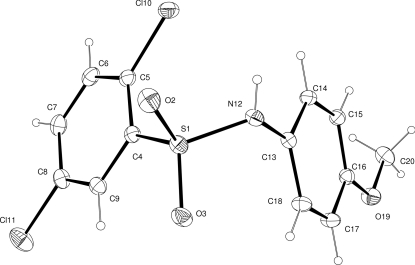
The molecular structure of (I) showing the atom-numbering scheme and 30% probability ellipsoids.

**Fig. 2.**
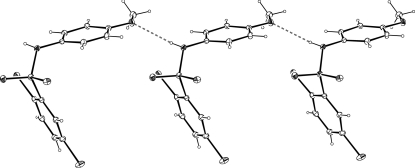
Part of the crystal structure of (I), showing chain of molecules linked by intermolecular N—H···O hydrogen bonds (dashed lines) along the b axis.

## supplementary crystallographic information

### Comment

In continuation of our studies of sulfonamides synthesis (Khan *et al.*, 2010; Sharif *et al.*, 2010), of interest owing to their biological properties (Korolkovas, 1988; Mandell & Sande, 1992). Herein, the crystal structure of (I) is described

In the title compound (I), (Fig. 1), the 4-methoxyphenyl moiety is almost planar with r.m.s. deviation of 0.018 Å from the corresponding least-squares plane defined by the nine constituent atoms. The dihedral angle between the benzene rings is 74.37 (3) °. In the crystal, intermolecular N—H···O hydrogen bonds link the molecules into chains along the *b* axis (Table 1, Fig. 2).

### Experimental

To *para*-anisidine (123 mg, 1 mmol) in distilled water (10 ml) was added 2,5-dichloro benzene sulfonyl chloride (0.245 mg, 1 mmol) with stirring at room temperature while maintaining the pH = 8 using 3% sodium carbonate. The progress of the reaction was monitored by TLC. The precipitate formed in this way was washed with water, dried and crystallized from methanol.

### Refinement

The H atom of the NH group was located in a difference Fourier map and refined freely. The remaining H atoms were positioned geometrically and refined using a riding model with C—H = 0.93–0.96 Å, and with *U*_iso_(H) = 1.2*U*_eq_ (C) for aromatic and 1.5*U*_eq_(C) for methyl H atoms.

### Crystal data

**Table:**

C_13_H_11_Cl_2_NO_3_S	*F*(000) = 680
*M**_r_* = 332.19	*D*_x_ = 1.582 Mg m^−^^3^
Monoclinic, *P*2_1_/*c*	Mo *K*α radiation, λ = 0.71073 Å
Hall symbol: -P 2ybc	Cell parameters from 4792 reflections
*a* = 13.1599 (4) Å	θ = 2.9–28.0°
*b* = 7.8179 (2) Å	µ = 0.62 mm^−^^1^
*c* = 14.4830 (5) Å	*T* = 296 K
β = 110.566 (1)°	Block, colourless
*V* = 1395.09 (7) Å^3^	0.25 × 0.17 × 0.12 mm
*Z* = 4	

### Data collection

**Table:**

Bruker APEXII CCD diffractometer	*R*_int_ = 0.030
φ and ω scans	θ_max_ = 28.3°, θ_min_ = 3.0°
13132 measured reflections	*h* = −17→17
3456 independent reflections	*k* = −10→10
2690 reflections with *I* > 2σ(*I*)	*l* = −18→19

### Refinement

**Table:**

Refinement on *F*^2^	0 restraints
Least-squares matrix: full	H atoms treated by a mixture of independent and constrained refinement
*R*[*F*^2^ > 2σ(*F*^2^)] = 0.039	*w* = 1/[σ^2^(*F*_o_^2^) + (0.045*P*)^2^ + 0.6614*P*] where *P* = (*F*_o_^2^ + 2*F*_c_^2^)/3
*wR*(*F*^2^) = 0.108	(Δ/σ)_max_ = 0.001
*S* = 1.06	Δρ_max_ = 0.46 e Å^−^^3^
3456 reflections	Δρ_min_ = −0.31 e Å^−^^3^
186 parameters	

### Special details

**Table:**

Geometry. All e.s.d.'s (except the e.s.d. in the dihedral angle between two l.s. planes) are estimated using the full covariance matrix. The cell e.s.d.'s are taken into account individually in the estimation of e.s.d.'s in distances, angles and torsion angles; correlations between e.s.d.'s in cell parameters are only used when they are defined by crystal symmetry. An approximate (isotropic) treatment of cell e.s.d.'s is used for estimating e.s.d.'s involving l.s. planes.

### Fractional atomic coordinates and isotropic or equivalent isotropic displacement parameters (Å^2^)

**Table:**

	*x*	*y*	*z*	*U*_iso_*/*U*_eq_	
S1	0.72780 (4)	0.85926 (6)	0.82650 (3)	0.03916 (14)	
O2	0.73299 (14)	1.04147 (19)	0.82443 (12)	0.0552 (4)	
O3	0.69500 (12)	0.7655 (2)	0.73648 (10)	0.0493 (4)	
C4	0.85981 (16)	0.7804 (2)	0.89567 (14)	0.0369 (4)	
C5	0.91579 (17)	0.8160 (3)	0.99528 (15)	0.0419 (4)	
C6	1.02137 (18)	0.7615 (3)	1.03996 (17)	0.0509 (5)	
H6	1.0576	0.7843	1.1065	0.061*	
C7	1.07387 (19)	0.6737 (3)	0.98749 (18)	0.0539 (6)	
H7	1.1455	0.6385	1.0176	0.065*	
C8	1.01785 (18)	0.6389 (3)	0.88887 (17)	0.0485 (5)	
C9	0.91210 (17)	0.6898 (3)	0.84328 (15)	0.0427 (4)	
H9	0.8756	0.6635	0.7772	0.051*	
Cl10	0.85599 (5)	0.93075 (8)	1.06480 (4)	0.05536 (17)	
Cl11	1.08144 (6)	0.53037 (11)	0.82003 (6)	0.0786 (2)	
N12	0.64500 (14)	0.8123 (2)	0.88365 (13)	0.0398 (4)	
H12	0.6441 (19)	0.888 (3)	0.9181 (17)	0.046 (7)*	
C13	0.63424 (15)	0.6413 (2)	0.91467 (13)	0.0350 (4)	
C14	0.65129 (17)	0.6093 (3)	1.01225 (14)	0.0412 (4)	
H14	0.6706	0.6986	1.0575	0.049*	
C15	0.64003 (17)	0.4456 (3)	1.04402 (15)	0.0423 (5)	
H15	0.6504	0.4257	1.11	0.051*	
C16	0.61338 (16)	0.3123 (3)	0.97760 (15)	0.0405 (4)	
C17	0.59349 (19)	0.3448 (3)	0.87865 (15)	0.0476 (5)	
H17	0.5737	0.2557	0.8333	0.057*	
C18	0.60287 (18)	0.5083 (3)	0.84720 (15)	0.0448 (5)	
H18	0.5881	0.5295	0.7806	0.054*	
O19	0.60296 (14)	0.1448 (2)	1.00210 (12)	0.0560 (4)	
C20	0.6237 (2)	0.1095 (3)	1.10353 (19)	0.0610 (6)	
H20A	0.6977	0.138	1.1414	0.091*	
H20B	0.6117	−0.0098	1.1116	0.091*	
H20C	0.5759	0.1765	1.126	0.091*	

### Atomic displacement parameters (Å^2^)

**Table:**

	*U*^11^	*U*^22^	*U*^33^	*U*^12^	*U*^13^	*U*^23^
S1	0.0448 (3)	0.0397 (3)	0.0347 (2)	0.0049 (2)	0.0162 (2)	0.00713 (19)
O2	0.0669 (10)	0.0399 (8)	0.0638 (10)	0.0066 (7)	0.0292 (9)	0.0140 (7)
O3	0.0502 (8)	0.0672 (10)	0.0303 (7)	0.0043 (7)	0.0137 (6)	0.0024 (7)
C4	0.0414 (10)	0.0341 (9)	0.0361 (9)	−0.0023 (8)	0.0147 (8)	0.0044 (7)
C5	0.0504 (11)	0.0387 (10)	0.0371 (10)	−0.0093 (9)	0.0158 (9)	0.0011 (8)
C6	0.0489 (12)	0.0532 (13)	0.0423 (11)	−0.0122 (10)	0.0056 (10)	0.0042 (10)
C7	0.0414 (11)	0.0580 (14)	0.0583 (14)	−0.0021 (10)	0.0128 (10)	0.0121 (11)
C8	0.0474 (12)	0.0490 (12)	0.0533 (12)	0.0055 (9)	0.0231 (10)	0.0097 (10)
C9	0.0471 (11)	0.0426 (11)	0.0404 (10)	0.0018 (9)	0.0178 (9)	0.0049 (8)
Cl10	0.0707 (4)	0.0531 (3)	0.0446 (3)	−0.0098 (3)	0.0231 (3)	−0.0122 (2)
Cl11	0.0661 (4)	0.1040 (6)	0.0740 (5)	0.0343 (4)	0.0349 (4)	0.0095 (4)
N12	0.0480 (10)	0.0371 (9)	0.0386 (9)	0.0037 (7)	0.0205 (8)	−0.0011 (7)
C13	0.0340 (9)	0.0368 (10)	0.0363 (9)	0.0017 (7)	0.0151 (8)	−0.0002 (8)
C14	0.0475 (11)	0.0427 (11)	0.0356 (10)	−0.0059 (8)	0.0175 (9)	−0.0070 (8)
C15	0.0475 (11)	0.0487 (12)	0.0332 (10)	−0.0065 (9)	0.0172 (9)	−0.0002 (8)
C16	0.0415 (10)	0.0391 (10)	0.0427 (11)	−0.0018 (8)	0.0171 (9)	0.0011 (8)
C17	0.0629 (13)	0.0414 (11)	0.0390 (11)	−0.0044 (9)	0.0185 (10)	−0.0090 (9)
C18	0.0574 (13)	0.0449 (11)	0.0312 (9)	−0.0003 (9)	0.0146 (9)	−0.0023 (8)
O19	0.0776 (11)	0.0403 (8)	0.0550 (9)	−0.0060 (7)	0.0290 (8)	0.0021 (7)
C20	0.0800 (17)	0.0519 (14)	0.0613 (15)	0.0073 (12)	0.0377 (13)	0.0149 (12)

### Geometric parameters (Å, °)

**Table:**

S1—O3	1.4243 (15)	N12—H12	0.78 (2)
S1—O2	1.4269 (16)	C13—C14	1.374 (3)
S1—N12	1.6250 (17)	C13—C18	1.387 (3)
S1—C4	1.783 (2)	C14—C15	1.386 (3)
C4—C9	1.385 (3)	C14—H14	0.93
C4—C5	1.398 (3)	C15—C16	1.377 (3)
C5—C6	1.378 (3)	C15—H15	0.93
C5—Cl10	1.731 (2)	C16—O19	1.376 (2)
C6—C7	1.377 (3)	C16—C17	1.387 (3)
C6—H6	0.93	C17—C18	1.377 (3)
C7—C8	1.385 (3)	C17—H17	0.93
C7—H7	0.93	C18—H18	0.93
C8—C9	1.373 (3)	O19—C20	1.424 (3)
C8—Cl11	1.732 (2)	C20—H20A	0.96
C9—H9	0.93	C20—H20B	0.96
N12—C13	1.433 (3)	C20—H20C	0.96
			
O3—S1—O2	119.74 (10)	S1—N12—H12	108.5 (18)
O3—S1—N12	107.95 (10)	C14—C13—C18	119.27 (18)
O2—S1—N12	106.36 (10)	C14—C13—N12	119.59 (17)
O3—S1—C4	104.98 (9)	C18—C13—N12	121.09 (17)
O2—S1—C4	108.22 (10)	C13—C14—C15	120.78 (18)
N12—S1—C4	109.34 (9)	C13—C14—H14	119.6
C9—C4—C5	118.95 (19)	C15—C14—H14	119.6
C9—C4—S1	116.27 (15)	C16—C15—C14	119.84 (18)
C5—C4—S1	124.56 (16)	C16—C15—H15	120.1
C6—C5—C4	120.1 (2)	C14—C15—H15	120.1
C6—C5—Cl10	118.46 (17)	O19—C16—C15	124.31 (18)
C4—C5—Cl10	121.40 (16)	O19—C16—C17	116.17 (18)
C7—C6—C5	120.9 (2)	C15—C16—C17	119.51 (19)
C7—C6—H6	119.5	C18—C17—C16	120.37 (19)
C5—C6—H6	119.5	C18—C17—H17	119.8
C6—C7—C8	118.6 (2)	C16—C17—H17	119.8
C6—C7—H7	120.7	C17—C18—C13	120.14 (18)
C8—C7—H7	120.7	C17—C18—H18	119.9
C9—C8—C7	121.4 (2)	C13—C18—H18	119.9
C9—C8—Cl11	118.56 (18)	C16—O19—C20	116.76 (18)
C7—C8—Cl11	120.00 (18)	O19—C20—H20A	109.5
C8—C9—C4	120.0 (2)	O19—C20—H20B	109.5
C8—C9—H9	120	H20A—C20—H20B	109.5
C4—C9—H9	120	O19—C20—H20C	109.5
C13—N12—S1	121.87 (14)	H20A—C20—H20C	109.5
C13—N12—H12	119.1 (18)	H20B—C20—H20C	109.5

### Hydrogen-bond geometry (Å, °)

**Table:**

*D*—H···*A*	*D*—H	H···*A*	*D*···*A*	*D*—H···*A*
N12—H12···O19^i^	0.78 (2)	2.50 (3)	3.267 (2)	168 (2)

Symmetry codes: (i) *x*, *y*+1, *z*.
